# Midazolam suppresses interleukin-1β-induced interleukin-6 release from rat glial cells

**DOI:** 10.1186/1742-2094-8-68

**Published:** 2011-06-17

**Authors:** Kumiko Tanabe, Osamu Kozawa, Hiroki Iida

**Affiliations:** 1 Department of Anesthesiology and Pain Medicine, Gifu University Graduate School of Medicine, Gifu 501-1194, Japan; 2 Department of Pharmacology, Gifu University Graduate School of Medicine, Gifu 501-1194, Japan

## Abstract

**Background:**

Peripheral-type benzodiazepine receptor (PBR) expression levels are low in normal human brain, but their levels increase in inflammation, brain injury, neurodegenerative states and gliomas. It has been reported that PBR functions as an immunomodulator. The mechanisms of action of midazolam, a benzodiazepine, in the immune system in the CNS remain to be fully elucidated. We previously reported that interleukin (IL)-1β stimulates IL-6 synthesis from rat C6 glioma cells and that IL-1β induces phosphorylation of inhibitory kappa B (IκB), p38 mitogen-activated protein (MAP) kinase, stress-activated protein kinase (SAPK)/c-*Jun *N-terminal kinase (JNK), extracellular signal-regulated kinase 1/2, and signal transducer and activator of transcription (STAT)3. It has been shown that p38 MAP kinase is involved in IL-1β-induced IL-6 release from these cells. In the present study, we investigated the effect of midazolam on IL-1β-induced IL-6 release from C6 cells, and the mechanisms of this effect.

**Methods:**

Cultured C6 cells were stimulated by IL-1β. IL-6 release from C6 cells was measured using an enzyme-linked immunosorbent assay, and phosphorylation of IκB, the MAP kinase superfamily, and STAT3 was analyzed by Western blotting.

**Results:**

Midazolam, but not propofol, inhibited IL-1β-stimulated IL-6 release from C6 cells. The IL-1β-stimulated levels of IL-6 were suppressed by wedelolactone (an inhibitor of IκB kinase), SP600125 (an inhibitor of SAPK/JNK), and JAK inhibitor I (an inhibitor of JAK 1, 2 and 3). However, IL-6 levels were not affected by PD98059 (an inhibitor of MEK1/2). Midazolam markedly suppressed IL-1β-stimulated STAT3 phosphorylation without affecting the phosphorylation of p38 MAP kinase, SAPK/JNK or IκB.

**Conclusion:**

These results strongly suggest that midazolam inhibits IL-1β-induced IL-6 release in rat C6 glioma cells via suppression of STAT3 activation. Midazolam may affect immune system function in the CNS.

## Background

Midazolam, a benzodiazepine, is used as an intravenously administered anesthetic for premedication, induction and maintenance of general anesthesia, and sedation in intensive care unit patients, who sometimes need treatment for central nervous system (CNS) diseases or who have CNS complications [1]. Benzodiazepine receptors consist of two types of receptors, central-type benzodiazepine receptors (CBRs), which are coupled to type A gamma-amminobutyric acid (GABA_A_) receptors, and peripheral-type benzodiazepine receptors (PBRs), which are not coupled to GABA_A _receptors [2,3]. Although it has been demonstrated that midazolam binds to and activates both CBRs and PBRs [4], midazolam has a hypnotic effect that is mediated via CBRs in neurons [1-3]. With regard to receptor expression, CBRs are expressed exclusively in the CNS [2]. Expression of CBRs coupled to GABA_A _receptors in astrocytes has been shown to be influenced by astrocytic maturation, differentiation, and activation [5]. In contrast, PBRs are detected in many peripheral tissues and cells, such as kidney, endocrine organs and monocytes [2,3]. While the expression levels of PBRs are low in normal human brain, levels in both astrocytes and microglia increase in conditions of glial activation; for example, inflammation, brain injury, neurodegenerative states, and gliomas [2,3]. C6 cells, derived from rat glioma cells, have been shown to express PBRs and few CBRs [6]. These cells are thus suitable for investigations of PBR functions in astrocytes.

PBRs have been reported to function in the regulation of cellular proliferation, immunomodulation, steroidogenesis, oxidative processes, and programmed cell death [2,3]. Several animal studies have demonstrated that midazolam can improve neural recovery after anoxia and ischemia [7]. Cytokines, particularly interleukin (IL)-1β and tumor necrosis factor (TNF)-α, activate the immune system and enhance brain damage [8]. Midazolam has been shown to inhibit IL-6 mRNA expression in human peripheral blood mononuclear cells [9], and to suppress lipopolysachccaride (LPS)-induced nitric oxide and TNF-α release from rat microglia via PBRs [10]. Thus, these results led us to speculate that midazolam might modulate immune system function in the CNS. However, the exact mechanism of action of midazolam effects on immune system in the CNS remain to be fully elucidated.

In the physiological CNS, IL-1β, a pro-inflammatory cytokine, is expressed at low levels [11-13]. IL-1 plays a role in some physiological processes including sleep and synaptic plasticity [12]. Levels of IL-1β increase in cerebrospinal fluid in patients with traumatic brain injury, stroke and neurodegenerative diseases [12]. The main source of brain IL-1β after acute insult is microglia [11-13]. Astrocytes also produce IL-1β in response to such stimuli, with a time course slightly later than that of microglia [11-13]. IL-1β induces the production of other cytokines, such as TNF-α and IL-6, from microglia and astrocytes [12,13]. We have previously reported that IL-1β significantly induces IL-6 synthesis in C6 glioma cells [14]. Cytokines like IL-1β and IL-6 have been implicated in neuroinflammation, astrogliosis, brain ischemia and chronic CNS diseases [11-13,15]. In the present study, we investigated the effect of midazolam on IL-1β-induced IL-6 release from C6 cells, and the mechanisms underlying this effect.

## Methods

### Materials

IL-6 enzyme-linked immunosorbent assay (ELISA) kits and IL-1β were obtained from R&D System (Minneapolis, MN). Midazolam and propofol were obtained from Sigma-Aldrich Chemical Co. (St. Louis, MO). Wedelolactone, SP600125, PD98059 and Janus family of tyrosine kinase (JAK) inhibitor I were obtained from Calbiochem-Novabiochem Co. (La Jolla, CA). Phospho-specific p38 mitogen-activated protein (MAP) kinase, p38 MAP kinase, phospho-specific stress-activated protein kinase/c-*Jun *N-terminal kinase (SAPK/JNK), SAPK/JNK, phospho-specific inhibitory kappa B (IκB), IκB, phospho-specific signal transducer and activator of transcription (STAT)3 and STAT3 antibodies were purchased from Cell Signaling (Beverly, MA). Glyceraldehyde-3-phosphate dehydrogenase (GAPDH) antibodies were purchased from Santa Cruz Biotechnology, Inc. (Santa Cruz, CA). An enhanced chemiluminescence Western blotting detection system was obtained from GE Healthcare UK. Ltd. (Buckinghamshire, England). Other materials and chemicals were obtained from commercial sources. Wedelolactone, SP600125, PD98059 and JAK inhibitor I were dissolved in dimethyl sulfoxide. Propofol was dissolved in ethanol. The maximum concentration of dimethyl sulfoxide or ethanol was 0.1%, which did not affect the assay for IL-6 or Western blot analysis. The viability of cells with 0.1% dimethyl sulfoxide or ethanol treatment after 36 h was above 97% compared to the cells without treatment by trypan blue staining.

### Cell culture

Rat C6 glioma cells, obtained from the American Type Culture Collection (Rockville, MD), were seeded into 35-mm (5 × 10^4 ^cells/dish) or 90-mm (2 × 10^5 ^cells/dish) diameter dishes and maintained in Dulbecco's modified Eagle's medium (DMEM) containing 10% fetal bovine serum at 37°C in a humidified atmosphere of 5% CO_2_/95% air. The medium was exchanged for serum-free DMEM after 6 days. The cells were then used for experiments after 24 h. The cells were pretreated with midazolam, propofol, wedelolactone, SP600125, PD98059 or JAK inhibitor I for 60 min before IL-1β stimulation when indicated.

### Assay for IL-6

Cultured cells (35-mm diameter dishes) were stimulated with 10 ng/ml IL-1β in serum-free DMEM for 36 h. The conditioned medium was collected at the end of the incubation, and IL-6 concentration was measured using an ELISA kit. The absorbance of each sample at 450 nm and 540 nm was measured with a Multiscan JX ELISA reader (Thermo Labsystems, Helsinki, Finland). Absorbance was corrected with reference to a standard curve.

### Western blot analysis

Cultured cells (90-mm diameter dishes) were stimulated with 10 ng/ml IL-1β in serum-free DMEM for the indicated periods. The cells were washed twice with phosphate-buffered saline and then lysed and sonicated in a lysis buffer containing 62.5 mM Tris/HCl (pH 6.8), 2% sodium dodecyl sulfate (SDS), 50 mM dithiothreitol, and 10% glycerol. The sample was used for Western blot analysis. The samples were separated by SDS-polyacrylamide gel electrophoresis using the method of Laemmli [16] in 10% polyacrylamide gels. Western blot analysis was performed using phospho-specific p38 MAP kinase antibodies, p38 MAP kinase antibodies, phospho-specific SAPK/JNK antibodies, SAPK/JNK antibodies, phospho-specific IκB antibodies, IκB antibodies, phospho-specific STAT3 antibodies, STAT3 antibodies or GAPDH antibodies, with peroxidase-labeled antibodies raised in goat against rabbit IgG being used as secondary antibodies. Peroxidase activity on polyvinylidene difluoride membrane was visualized on X-ray film by utilizing an enhanced chemiluminescence Western blotting detection system.

### Statistical analysis

The data were analyzed by ANOVA followed by Bonferroni's method for multiple comparisons between pairs. *P *< 0.05 was considered to be significant. All data are presented as the mean ± SD of triplicate determinations. Each experiment was repeated three times with similar results.

## Results

### Effects of midazolam or propofol on IL-1β-induced IL-6 release from C6 cells

**Figure 1:**
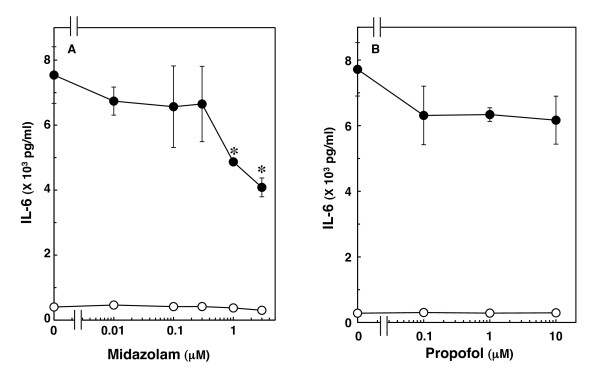
**Effects of midazolam or propofol on IL-1β-induced IL-6 release**. Cultured cells were pretreated with various concentrations of midazolam (A) or propofol (B) for 60 min, and then stimulated with 10 ng/ml IL-1β (closed circle) or vehicle (open circle) for 36 h in the presence of midazolam or propofol. Each value represents the mean ± SD of triplicate independent determinations of a representative experiment carried out three times. Similar results were obtained with two additional and different cell preparations. *P < 0.05 in comparison to the value of IL-1β alone.

We previously reported that IL-1β significantly induces IL-6 mRNA expression and stimulates IL-6 release in C6 glioma cells [14]. Midazolam, which by itself had little effect on IL-6 levels, significantly suppressed IL-1β-induced IL-6 release. The suppressive effect was concentration-dependent between 0.3 and 3 μM (Figure 1A). Midazolam (3 μM) caused a 47% inhibition of the IL-1β effect on IL-6 release. On the other hand, propofol, another intravenous anesthetic, did not affect IL-1β-induced IL-6 release at concentrations up to 10 μM (Figure 1B).

### Effect of wedelolactone on IL-1β-induced IL-6 release from C6 cells

**Figure 2:**
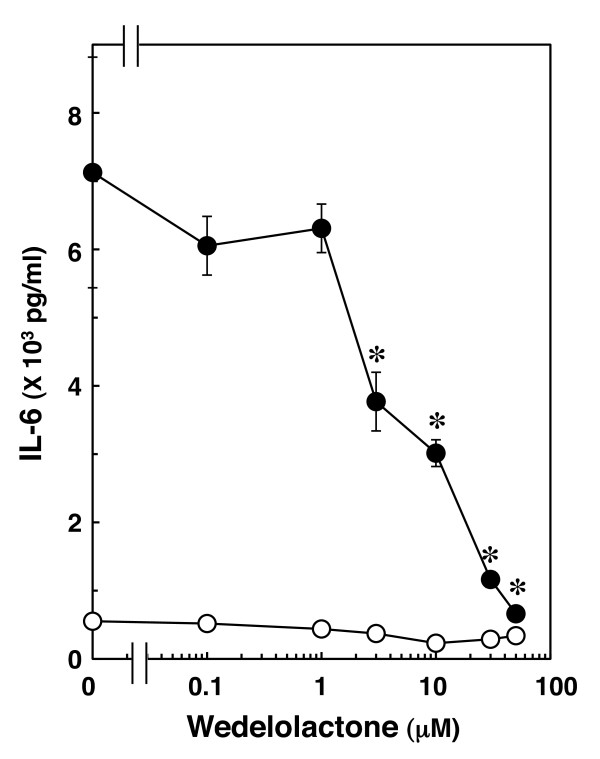
**Effect of wedelolactone on IL-1β-induced IL-6 release**. Cultured cells were pretreated with various concentrations of wedelolactone for 60 min, and then stimulated with 10 ng/ml IL-1β (closed circle) or vehicle (open circle) for 36 h in the presence of wedelolactone. Each value represents the mean ± SD of triplicate independent determinations of a representative experiment carried out three times. Similar results were obtained with two additional and different cell preparations. *P < 0.05 in comparison to the value of IL-1β alone.

It is well known that cytokines induce activation of the IκB-nuclear factor kappa B (NFκB) pathway after binding to receptors [11-13]. NFκB binds to its consensus sequence on a target gene promoting transcription and upregulation of gene expression in the nucleus [17]. We confirmed that IL-1β induces IκB phosphorylation and its degradation in a time-dependent manner in C6 cells [18]. Then, we next examined whether the IκB-NFκB pathway is involved in IL-1β-induced IL-6 release. Wedelolactone, an inhibitor of IκB kinase (IKK) [19], suppressed both IL-1β-induced phosphorylation and degradation of IκB at 50 μM in C6 cells [18]. Wedelolactone, which alone had little effect on the IL-6 levels, significantly inhibited IL-1β-induced IL-6 release. The suppressive effect of wedelolactone was concentration-dependent in the range between 1 and 50 μM (Figure 2).

### Effects of SP600125 or PD98059 on IL-1β-induced IL-6 release from C6 cells

**Figure 3:**
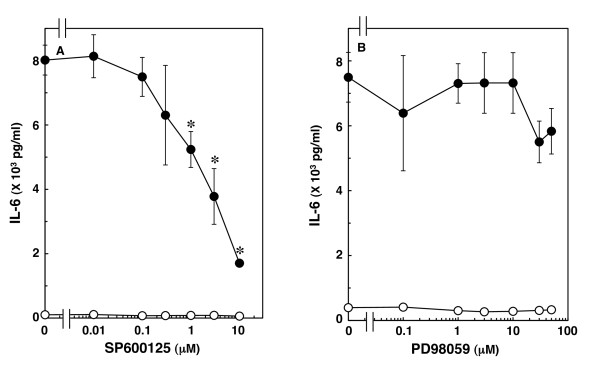
**Effects of SP600125 or PD98059 on IL-1β-induced IL-6 release**. Cultured cells were pretreated with various concentrations of SP600125 (A) or PD98059 (B) for 60 min, and then stimulated with 10 ng/ml IL-1β (closed circle) or vehicle (open circle) for 36 h in the presence of SP600125 or PD98059. Each value represents the mean ± SD of triplicate independent determinations of a representative experiment carried out three times. Similar results were obtained with two additional and different cell preparations. *P < 0.05 in comparison to the value of IL-1β alone.

MAP kinase superfamily members such as p38 MAP kinase, SAPK/JNK and extracellular signal-regulated kinase (Erk) 1/2 are central elements used by mammalian cells to transduce the various messages of variety of agonists [20]. We previously reported that IL-1β significantly induces the activation of p38 MAP kinase, SAPK/JNK and Erk 1/2 in C6 glioma cells [18]. SB203580, a specific inhibitor of p38 MAP kinase [21], suppresses IL-1β-induced IL-6 release from C6 cells, suggesting that p38 MAP kinase regulates IL-6 release [22]. To clarify whether other MAP kinases are involved in IL-1β-induced IL-6 release from these cells, we examined the effects of two MAP kinase inhibitors on IL-1β-induced IL-6 release. IL-6 release induced by IL-1β was markedly suppressed by SP600125, a specific inhibitor of SAPK/JNK [23], which alone had little effect on IL-6 levels (Figure 3A). The suppressive effect of SP600125 was concentration dependent between 0.3 and 10 μM. We found that SP600125 (10 μM) remarkably attenuated IL-1β-induced phosphorylation of SAPK/JNK [18]. However, PD98059, a specific inhibitor of upstream kinase (MEK1/2) that activates Erk 1/2 [24], failed to affect IL-1β-induced IL-6 release up to 50 μM (Figure 3B). We have previously confirmed that 10 μM PD98059 truly suppresses IL-1β-induced phosphorylation of Erk 1/2 in C6 cells [18].

### Effect of JAK inhibitor I on IL-1β-induced IL-6 release from C6 cells

**Figure 4:**
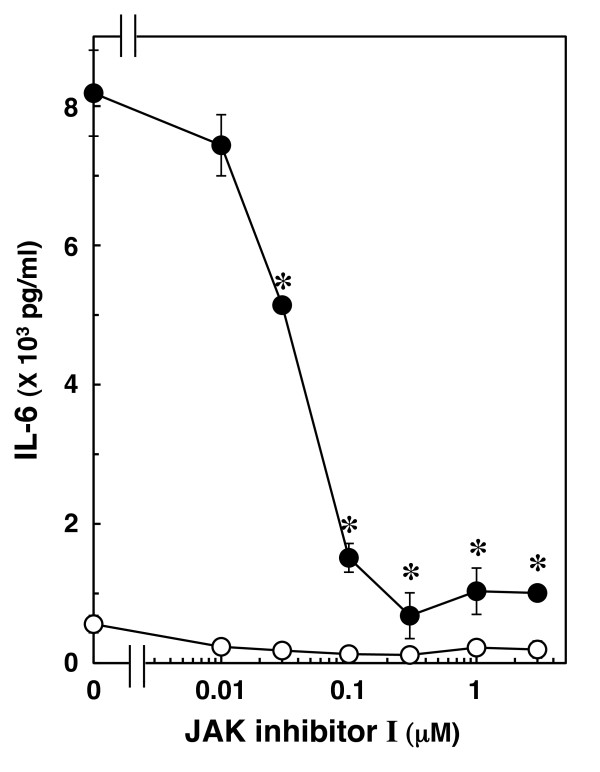
**Effect of JAK inhibitor I on IL-1β-induced IL-6 release**. Cultured cells were pretreated with various concentrations of JAK inhibitor I for 60 min, and then stimulated with 10 ng/ml IL-1β (closed circle) or vehicle (open circle) for 36 h in the presence of JAK inhibitor I. Each value represents the mean ± SD of triplicate independent determinations of a representative experiment carried out three times. Similar results were obtained with two additional and different cell preparations. *P < 0.05 in comparison to the value of IL-1β alone.

The JAK-STAT pathway has an essential role in driving a variety of biological responses to cytokines [25,26]. We previously reported that IL-1β induces activation of STAT3 in C6 glioma cells [18]. In order to clarify whether STAT3 is involved in IL-1β-induced IL-6 release in C6 cells, we examined the effect of JAK inhibitor I, an inhibitor of JAK 1, 2 and 3 [27], on IL-1β-induced IL-6 release. JAK inhibitor I, which by itself had little effect on the IL-6 levels, significantly suppressed IL-1β-induced IL-6 release. The effect of JAK inhibitor I was concentration dependent between 10 nM and 30 μM (Figure 4). In a previous study [18], we found that JAK inhibitor I (1 μM) truly reduces IL-1β-induced phosphorylation of STAT3 in C6 cells.

### Effects of midazolam on IL-1β-induced phosphorylation of IκB, p38 MAP kinase, SAPK/JNK, and STAT3 in C6 cells

**Figure 5:**
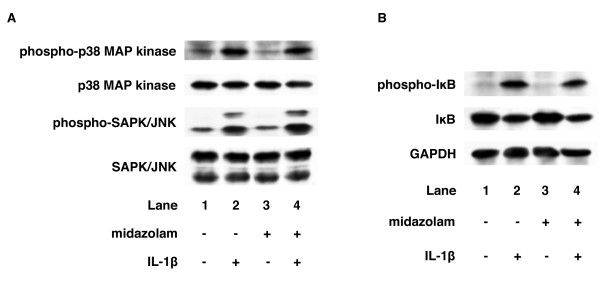
**Effects of midazolam on IL-1β-induced IκB, p38 MAP kinase or SAPK/JNK phosphorylation**. Cultured cells were pretreated with 10 μM midazolam for 60 min, and then stimulated with 10 ng/ml IL-1β or vehicle for 20 min (A) or 30 min (B) in the presence of midazolam. Cell extracts were analyzed by Western blotting using antibodies against phospho-specific IκB, IκB, GAPDH, phospho-specific p38 MAP kinase, p38 MAP kinase, phospho-specific SAPK/JNK or SAPK/JNK. Similar results were obtained with two additional and different cell preparations.

**Figure 6:**
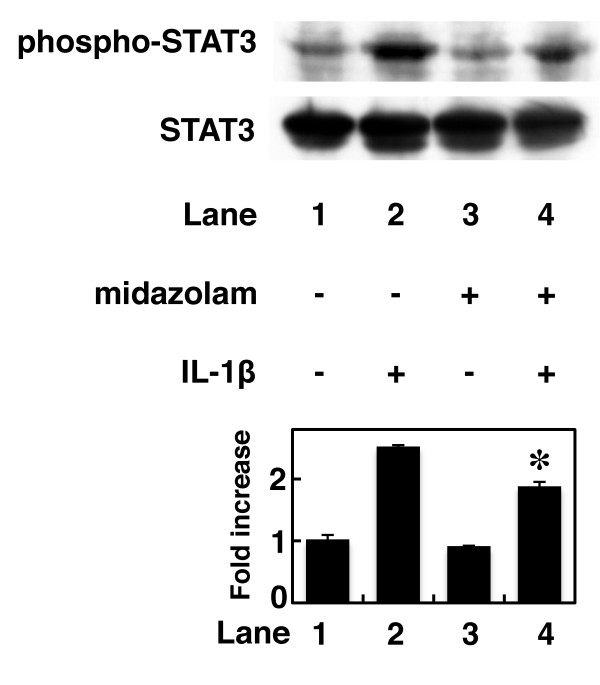
**Effect of midazolam on IL-1β-induced STAT3 phosphorylation**. Cultured cells were pretreated with 10 μM midazolam for 60 min, and then stimulated with 10 ng/ml IL-1β or vehicle for 60 min in the presence of midazolam. Cell extracts were analyzed by Western blotting using antibodies against phospho-specific STAT3 or STAT3. The histogram shows quantitative representations of the levels of IL-1β-induced phosphorylation of STAT3 obtained from laser densitometric analysis of three independent experiments, respectively. Similar results were obtained with two additional and different cell preparations. *P < 0.05 in comparison to the value of IL-1β alone.

In the present study, our results suggest that IL-1β induces IL-6 release through the IκB-NFκB pathway, p38 MAP kinase, SAPK/JNK and JAK-STAT3 pathway in C6 glioma cells. Finally, we investigated the action point of midazolam in IL-1β-stimulated IL-6 release from C6 cells. Midazolam failed to affect IL-1β-induced IκB, p38 MAP kinase or SAPK/JNK phosphorylation in C6 cells (Figure 5). In contrast, midazolam (10 μM) significantly inhibited IL-1β-induced STAT3 phosphorylation (Figure 6). Midazolam (10 μM) caused a 40% inhibition of the IL-1β effect on STAT3 phosphorylation.

## Discussion

In the present study, we find that midazolam significantly suppresses IL-1β-induced IL-6 release from C6 glioma cells. However, propofol, another intravenous anesthetic, failed to affect IL-1β-induced IL-6 release from C6 cells. Then, we next investigated the mechanisms of IL-1β-induced IL-6 release from C6 cells. IL-1β binds its receptor, which associates with IL-1 receptor-accessory proteins to initiate an intracellular signaling [11-13]. NFκB and the MAP kinase superfamily, including p38 MAP kinase, Erk 1/2 and SAPK/JNK, are then activated by IL-1β [11-13]. IκB is phosphorylated and degradated by IKK, and subsequently NFκB is freed from IκB and translocates into the nucleus [17]. In addition, the JAK-STAT pathway is recognized to have an important role in signaling of cytokines such as the interleukins [25,26]. Activation of the JAK-STAT pathway leads to rapid signaling from the cell surface to the nucleus [25,26]. We previously reported that IL-1β induces activation of IκB, p38 MAP kinase, SAPK/JNK, Erk 1/2 and STAT3 in C6 glioma cells [18]. SB203580, a specific inhibitor of p38 MAP kinase [21], reportedly reduces IL-1β-induced IL-6 release from C6 cells [22]. In the present study, IL-6 release induced by IL-1β was suppressed by wedelolactone, an inhibitor of IKK [19], by SP600125, a specific inhibitor of SAPK/JNK [23], and by JAK inhibitor I, an inhibitor of JAK 1, 2 and 3 [27]; but not by PD98059, a specific inhibitor of MEK1/2 [24]. Therefore, based on these findings, it is probable that IL-1β induces IL-6 release through activation of the IκB-NFκB pathway, p38 MAP kinase, SAPK/JNK and JAK-STAT3 pathway in C6 glioma cells. These results are consistent with our previous report, in which we found that TNF-α induces IL-6 release through the IκB-NFκB pathway, p38 MAP kinase, SAPK/JNK and the JAK-STAT3 pathway in C6 cells [28]. In addition, we investigated which pathway is involved in IL-1β-induced IL-6 release suppression by midazolam. Midazolam markedly inhibited IL-1β-induced STAT3 phosphorylation. The inhibitory rate of IL-1β-induced IL-6 levels caused by midazolam was similar to the inhibitory rate of IL-1β-induced STAT3 phosphorylation. In contrast, IκB, p38 MAP kinase and SAPK/JNK phosphorylation were not affected by midazolam. Taking our findings into account, it is most likely that midazolam inhibits IL-1β-induced IL-6 release through the JAK/STAT3 pathway suppression in C6 glioma cells.

It has previously been reported that midazolam inhibits N-formyl-methionyl-leucyl-phenylalanine-induced p38 MAP kinase activation in neutrophils [29], thrombin-induced p38 MAP kinase activation in rat cardiac myocytes [30], and LPS-induced activation of IκB-NFκB pathway and p38 MAP kinase in a murine macrophage cell line [31]. In the present study, we show that midazolam significantly reduces IL-1β-induced STAT3 phosphorylation. Seven STAT proteins have been identified in mammalian cells [25,26]. In the CNS, STAT3 plays important roles in axonal regeneration and post-ischemic brain damage [32,33]. It has been reported that STAT3 activation is necessary for improved axonal regeneration in the spinal cord after injury [32] and that the suppression of STAT3 activation induced by brain ischemia in microglia prevents inflammation and brain damage [33]. It has been reported that olanzapine, one of the benzodiazepines, induces phosphorylation of STAT3 in a rat cortical cell line, resulting in desensitization of serotonin receptor signaling [34]. While midazolam binds to CBRs and PBRs [4], few CBRs are expressed in C6 cells [6]. It is known that PBRs are mainly located in the outer membrane of mitochondria [2]. Since mitochondria are the source and target of reactive oxygen species (ROS), it has been speculated that activation of PBRs suppresses ROS production and protects the CNS from ROS-induced damage [2]. It has recently been reported that a ROS scavenger inhibits STAT3 activation induced by cerebral ischemia/reperfusion damage in rats, reduces infarct size and improves neurological outcomes [35]. Based on these findings, it is possible that midazolam might inhibit IL-1β-induced STAT3 phosphorylation and IL-6 release through suppression of ROS production via PBRs. However, the role of STAT3 in benzodiazepine intracellular signaling in the CNS is not yet clarified. Further investigation will be required to clarify the significance of STAT3 in the CNS.

It is generally known that benzodiazepines modulate immune system [2,9,10]. In addition, benzodiazepines reportedly have neuroprotective effects, although to our knowledge there are no studies indicating better clinical outcomes [1]. IL-6 is well recognized as a pro-inflammatory cytokine and plays a key role in neuroinflammation [15]. Neuroinflammation accompanies neurodegenerative diseases and other brain disorders, such as, for instance, hypoxia/ischemia, traumatic brain injury, infections and epileptic seizures [15]. It is possible that midazolam might show neuroprotective effects through suppression of IL-6 levels in brain. In our present study, propofol, another intravenous anesthetic, failed to affect IL-1β-induced IL-6 release from C6 cells. Propofol is also suggested to affect the immune system [36,37] and to have neuroprotective effects in experimental conditions [38]. To the best of our knowledge, however, there are no reports regarding propofol effects on the CNS immune system. Based on our findings, it seems unlikely that the neuroprotective effect of propofol is due to suppression of IL-6 release from glial cells. IL-6 is expressed in the CNS in basal conditions, suggesting that IL-6 has a crucial role in normal physiological processes [15]. Recently, IL-6 has been reported to have anti-inflammatory properties in addition to pro-inflammatory roles in the CNS as follows: IL-6 enhances neuronal differentiation and promotes the survival of several types of neurons [15]. At the present time, IL-6 is considered to have both advantageous and disadvantageous effects in the CNS, and also to be a valid therapeutic target for the treatment of CNS disorders. Intravenous anesthetics are given to patients under various conditions such as brain injury, ischemia, neurodegenerative diseases and neuroinflammation. Midazolam might have an influence in patients with elevated IL-6 levels in the CNS; however, the exact clinical effects are not clear. It has been reported that blood concentrations of midazolam reach as much as 1.9 μM after a bolus injection [39]. The suppressive effect of midazolam on IL-6 release that we find in the present study was observed at concentrations over 0.3 μM. Therefore, it seems likely that immunomodulation of the CNS by midazolam might occur in clinical use. Further investigation is necessary to elucidate anesthetics' effects on immune system systemically or by organ, including the CNS.

In conclusion, our findings strongly suggest that midazolam inhibits IL-1β-induced IL-6 release in rat C6 glioma cells via suppression of STAT3 activation. It is possible that midazolam may affect immune system function in the CNS.

## Competing interests

The authors declare that they have no competing interests.

## Authors' contributions

KT and OK conceived the study, participated in its design and coordination, analyzed the data and drafted the manuscript. KT performed the experiments. HI provided useful advice. All authors read and approved the final manuscript.
